# Single response assessment of transplant-ineligible multiple myeloma: a supplementary analysis of JCOG1105 (JCOG1105S1)

**DOI:** 10.1093/jjco/hyab066

**Published:** 2021-05-06

**Authors:** Nobuhiko Nakamura, Dai Maruyama, Ryunosuke Machida, Tatsuo Ichinohe, Nobuyuki Takayama, Rie Ohba, Ken Ohmachi, Yoshitaka Imaizumi, Masahito Tokunaga, Hiroo Katsuya, Isao Yoshida, Kazutaka Sunami, Mitsutoshi Kurosawa, Nobuko Kubota, Hiroaki Morimoto, Miki Kobayashi, Harumi Kato, Yoshihiro Kameoka, Yoshitoyo Kagami, Masahiro Kizaki, Kazuto Takeuchi, Wataru Munakata, Shinsuke Iida, Hirokazu Nagai

**Affiliations:** Department of Hematology and Infectious Disease, Gifu University Hospital, Gifu, Japan; Department of Hematology Oncology, Cancer Institute Hospital of Japanese Foundation for Cancer Research, Tokyo, Japan; JCOG Data Center/Operations Office, National Cancer Center Hospital, Tokyo, Japan; Department of Hematology and Oncology, Hiroshima University Research Institute for Radiation Biology and Medicine, Hiroshima, Japan; Department of Hematology, Kyorin University School of Medicine, Tokyo, Japan; Department of Clinical Oncology and Hematology, The Jikei University Daisan Hospital, Tokyo, Japan; Department of Hematology/Oncology, Tokai University School of Medicine, Isehara, Japan; Department of Hematology, Nagasaki University Hospital, Nagasaki, Japan; Department of Hematology, Imamura General Hospital, Kagoshima, Japan; Division of Hematology, Respiratory Medicine and Oncology, Department of Internal Medicine, Faculty of Medicine, Saga University, Saga, Japan; Department of Hematologic Oncology, National Hospital Organization Shikoku Cancer Center, Matsuyama, Japan; Department of Hematology, National Hospital Organization Okayama Medical Center, Okayama, Japan; Department of Hematology, National Hospital Organization Hokkaido Cancer Center, Sapporo, Japan; Department of Hematology, Saitama Cancer Center, Saitama, Japan; Department of Hematology, University of Occupational and Environmental Health, Kitakyushu, Japan; Department of Hematology and Oncology, Japanese Red Cross Nagoya Daini Hospital, Nagoya, Japan; Department of Hematology and Cell Therapy, Aichi Cancer Center, Nagoya, Japan; Department of Hematology, Nephrology and Rheumatology, Akita University School of Medicine, Akita, Japan; Department of Hematology, Toyota Kosei Hospital, Toyota, Japan; Department of Hematology, Saitama Medical Center, Saitama Medical University, Kawagoe, Japan; First Department of Internal Medicine, Ehime University Hospital, Toon, Japan; Department of Hematology, National Cancer Center Hospital, Tokyo, Japan; Department of Hematology and Oncology, Nagoya City University Hospital, Nagoya, Japan; Department of Hematology, National Hospital Organization Nagoya Medical Center, Nagoya, Japan

**Keywords:** bortezomib, melphalan, multiple myeloma, response criteria

## Abstract

**Background:**

The International Myeloma Working Group response criteria require two consecutive assessments of paraprotein levels. We conducted an exploratory analysis to evaluate whether a single response assessment could be a substitute for the International Myeloma Working Group criteria using data from JCOG1105, a randomized phase II study on melphalan, prednisolone and bortezomib.

**Methods:**

Of 91 patients with transplant-ineligible newly diagnosed multiple myeloma, 79 patients were included. We calculated the kappa coefficient to evaluate the degree of agreement between the International Myeloma Working Group criteria and the single response assessment.

**Results:**

Based on the International Myeloma Working Group criteria, 11 (13.9%), 20 (25.3%), 36 (45.6%) and 12 (15.2%) patients had stringent complete response/complete response, very good partial response, partial response and stable disease, respectively. Based on the single response assessment, 17 (21.5%), 19 (24.1%), 35 (44.3%) and 8 (10.1%) patients had stringent complete response/complete response, very good partial response, partial response and stable disease, respectively. The kappa coefficient was 0.76 (95% confidence interval, 0.65–0.88), demonstrating good agreement. The single response assessment was not inferior to the International Myeloma Working Group criteria in the median progression-free survival (3.8 and 2.9 years) in stringent complete response/complete response patients, suggesting that the single response assessment was not an overestimation.

**Conclusions:**

The single response assessment could be a substitute for the current International Myeloma Working Group criteria for transplant-ineligible newly diagnosed multiple myeloma.

## Teaser

The single response assessment could be a substitute for the current International Myeloma Working Group criteria with two consecutive assessments in patients with transplant-ineligible newly diagnosed multiple myeloma.

## Introduction

Multiple myeloma (MM) is a malignant disorder characterized by a clonal proliferation of plasma cells producing a monoclonal immunoglobulin. The European Group for Blood and Bone Marrow Transplant/International Bone Marrow Transplant Registry/American Bone Marrow Transplant Registry (EBMT/IBMTR/ABMTR) published criteria for the response and progression of MM treated by stem cell transplantation, commonly referred to as the EBMT criteria (1). They defined complete response (CR), partial response (PR) and minimal response (MR), which required that the response was maintained for a minimum of 6 weeks to avoid recording a transient response. In 2006, the International Myeloma Working Group (IMWG) developed uniform response criteria, which have been used to measure the effect of treatment (2). All response categories require two consecutive assessments of serum or urine monoclonal protein concentrations at any time. Recently, IMWG has defined new response categories that also required two consecutive assessments of the paraprotein level (3). The purpose of these two consecutive assessments was to eliminate laboratory error or fluctuation of the measurement. However, two consecutive assessments are bothersome in clinical practice; furthermore, the interval between two assessments or exact timing of assessments is not clearly defined. Moreover, there is a risk of underestimating the best response due to the lack of a second response assessment, especially in the setting of clinical trials.

The Japan Clinical Oncology Group (JCOG)–Lymphoma Study Group (LSG) has conducted a randomized phase II study to optimize a more promising modified regimen containing melphalan, prednisolone and bortezomib (MPB) for transplant-ineligible newly diagnosed MM (TI-NDMM) (JCOG1105, jRCTs031180097) (4,5). The CR rate in this investigator-initiated study was lower than that in previous studies (6,7), and one possible reason for that difference was a failure to confirm CR with a second response assessment, including immunofixation electrophoresis of both serum and urine.

If a single response assessment can be demonstrated to be equally valid and precise as the current IMWG criteria, it could be used as a substitute for two consecutive assessments, therefore, lowering the burden on the medical system and avoiding the risk of underestimating the best response. Thus, this analysis aimed to evaluate whether a single response assessment can substitute the current IMWG criteria using data from JCOG1105.

**Figure 1.**
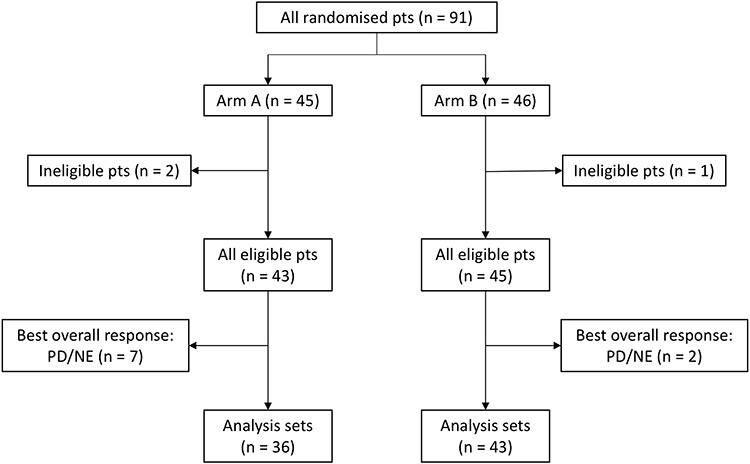
A patient-flow diagram for the supplementary analysis of data from JCOG1105. Arm A, one cycle of subcutaneous or intravenous bortezomib at 1.3 mg/m^2^ given twice weekly plus 9 mg/m^2^ of oral melphalan and 60 mg/m^2^ of prednisolone on days 1–4 in a 6-week cycle, followed by eight cycles of four weekly doses of bortezomib plus the same doses of melphalan and prednisolone in a 5-week cycle. Arm B, nine cycles of subcutaneous or intravenous bortezomib at 1.3 mg/m^2^ given in three weekly doses plus 7 mg/m^2^ of melphalan and 60 mg/m^2^ of oral prednisolone on days 1–4 in a 4-week cycle. NE, not evaluable; PD, progressive disease; pts, patients.

## Patients and methods

### Summary of JCOG1105

JCOG1105 (4,5) was a randomized phase II study to develop a more promising MPB regimen for the upcoming phase III study for TI-NDMM. The following patients were enrolled in JCOG1105 between July 2013 and April 2016: newly diagnosed symptomatic MM (IMWG 2003), ECOG performance status of 0–2 or 3 due to bone lesions, aged 65–79 years or 20–64 years, who were not candidates for stem cell transplantation, with preserved organ function. The follow-up ended in June 2019. The study protocol was approved by the Protocol Review Committee of JCOG and the respective institutional review boards. Informed consent about the secondary use of data was obtained from the enrolled patients upon registration in JCOG1105.

### Patients and response criteria

A total of 91 patients enrolled in JCOG1105 were analyzed in this study. Patients with the best overall response of progressive disease (PD) or not evaluable (NE) by the IMWG criteria were excluded. We excluded patients with PD because the IMWG criteria also allow for a single assessment to determine PD based on clinical judgment, and some patients can continue treatment because PD was not confirmed by two consecutive assessments. Two sets of response criteria were used in this supplementary analysis. The first one included IMWG criteria, which required two consecutive assessments made at any time (2) and adopted by JCOG1105. The second one was an exploratory criterion called a single response assessment, which did not require confirmation by the second assessment. The response subcategory was stringent complete response (sCR), CR, very good partial response (VGPR), PR and stable disease (SD). Because of the small number of patients with sCR, sCR and CR were combined into one response category.

### Statistical analysis

We analyzed the original data from JCOG1105 without collecting additional information. The primary endpoint of this supplementary analysis was the kappa coefficient to evaluate the degree of agreement between the IMWG criteria and the single response assessment using sCR/CR, VGPR, PR or SD. We selected the kappa coefficient, which is considered to be a more robust measure than a simple percentage calculation because it takes into account the possibility of chance agreement (8). The single response assessment was considered useful in cases with kappa coefficient of ≥0.7. The secondary endpoints were progression-free survival (PFS), overall survival (OS) and time to next treatment (TNT). Survival analysis in this study was performed to ensure whether the single response assessment was not inferior to the IMWG criteria due to the possibility of response overestimation. PFS, OS and TNT were estimated using the Kaplan-Meier method. The definitions of OS, PFS and TNT were identical to those reported in JCOG1105 as detailed in the previous study (4). OS, PFS and TNT were measured from the date of enrollment in JCOG1105 as described previously (4). All statistical analyses were performed by JCOG Data Center using SAS version 9.4 (SAS Institute, Cary, NC, USA).

## Results

### Patients characteristics

In JCOG1105, efficacy analyses were performed in all 88 eligible patients (4,5). Among them, nine patients with the best overall response of PD or NE by the IMWG criteria were excluded from this supplementary analysis. As a result, 79 patients were evaluated. Figure 1 shows the patient-flow diagram of this study. Patient characteristics are shown in Table 1. The median age was 72 (range, 65–79) years, and 48 patients (60.8%) were male. The number of patients with M-protein IgG, IgA, IgD and light chain was 48 (60.8%), 22 (27.8%), 1 (1.3%) and 8 (10.1%), respectively.

**Table 1:** Patient characteristics according to treatment arm

Characteristics	Arm A (*n* = 36)		Arm B (*n* = 43)		Total (*n* = 79)	
	No.	%	No.	%	No.	%
Age, years						
Median (range)	72 (65–79)	—	72 (65–78)	—	72 (65–79)	—
Sex						
Male	20	55.6	28	65.1	48	60.8
Female	16	44.4	15	34.9	31	39.2
ECOG PS						
0	18	50	19	44.2	37	46.8
1	12	33.3	15	34.9	27	34.2
2	2	5.6	2	4.7	4	5.1
3 (bone lesions)	4	11.1	7	16.3	11	13.9
M protein						
IgG	21	58.3	27	62.8	48	60.8
IgA	11	30.6	11	25.6	22	27.8
IgD	0	0	1	2.3	1	1.3
Light chain	4	11.1	4	9.3	8	10.1
ISS stage						
I	11	30.6	13	30.2	24	30.4
II	17	47.2	23	53.5	40	50.6
III	8	22.2	7	16.3	15	19
G-banded karyotype						
Normal	26	72.2	35	81.4	61	77.2
Abnormal	9	25	8	18.6	17	21.5
Not assessed	1	2.8	0	0	1	1.3
Chromosome translocation-associated gene expression (qRT-PCR)						
*CCND1*	12	—	18	—	30	—
*FGFR3*	6	—	1	—	7	—
*MAF*	1	—	0	—	1	—
Not expressed	9	—	10	—	19	—
Not assessed	9	—	14	—	23	—

ECOG PS, Eastern Cooperative Oncology Group Performance Status; ISS, International Staging System; qRT-PCR, quantitative reverse transcription polymerase chain reaction.

### Clinical response

Based on the IMWG criteria, 11 (13.9%), 20 (25.3%), 36 (45.6%) and 12 (15.2%) patients had sCR/CR, VGPR, PR and SD, respectively (Table 2). Based on the single response assessment, 17 (21.5%), 19 (24.1%), 35 (44.3%) and 8 (10.1%) patients had sCR/CR, VGPR, PR and SD, respectively. Four patients with VGPR and two with PR by the IMWG criteria had sCR/CR by the single response assessment. The single response assessment instead of the IMWG criteria upgraded the response levels in 13 patients (16.4%) (Supplementary Table S1). We did not include the two patients whose best response improved from CR to sCR in the analysis because we combined sCR and CR into one response category. The kappa coefficient between the IMWG criteria and the single response assessment was 0.76 [95% confidence interval (CI), 0.65–0.88], demonstrating good agreement.

**Table 2:** Clinical response based on IMWG criteria and single response assessment

	Single response assessment					
		sCR/CR	VGPR	PR	SD	Total (%)
	sCR/CR	11	0	0	0	11 (13.9)
	VGPR	4	16	0	0	20 (25.3)
IMWG criteria^a^	PR	2	3	31	0	36 (45.6)
	SD	0	0	4	8	12 (15.2)
	Total (%)	17 (21.5)	19 (24.1)	35 (44.3)	8 (10.1)	79

sCR, stringent complete response; CR, complete response; VGPR, very good partial response; PR, partial response; SD, stable disease; IMWG, International Myeloma Working Group; PD, progressive disease.

^a^All response categories require two consecutive assessments made at any time.

### Survival

**Figure 2.**
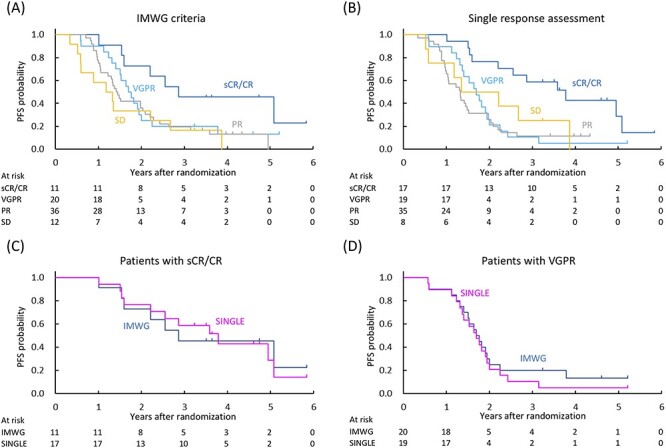
PFS curves by response status evaluated using the IMWG criteria (A) and the single response assessment (B). Patients with sCR/CR evaluated using the IMWG criteria (*n* = 11) and the single response assessment (*n* = 17) (C). Patients with VGPR evaluated using the IMWG criteria (*n* = 20) and the single response assessment (*n* = 19) (D). CR, complete response; IMWG, International Myeloma Working Group; sCR, stringent complete response; PFS, progression-free survival; VGPR, very good partial response.

At the data cut-off date (June 2019), the median follow-up of 79 patients was 3.9 years (range, 1.0–5.9). The 3-year PFS according to the IMWG criteria was 45.5% (95% CI, 16.7–70.7) for sCR/CR, 20.0% (95% CI, 6.2–39.3) for VGPR, 19.4% (95% CI, 8.6–33.6) for PR and 16.7% (95% CI, 2.7–41.3%) for SD (Fig. 2A). The 3-year PFS according to the single response assessment was 58.8% (95% CI, 32.5–77.8) for sCR/CR, 10.5% (95% CI, 1.8–28.4) for VGPR, 11.4% (95% CI, 3.6–24.2) for PR and 25.0% (95% CI, 3.7–55.8) for SD (Fig. 2B). The median PFS in sCR/CR patients was 2.9 years (95% CI, 1.5—not estimable) by the IMWG criteria and 3.8 years (95% CI, 1.6–5.1) by the single response assessment (Fig. 2C). The median PFS in VGPR patients was 1.7 years (95% CI, 1.3–2.0) by the IMWG criteria and 1.7 years (95% CI, 1.3–1.9) by the single response assessment (Fig. 2D).

**Figure 3.**
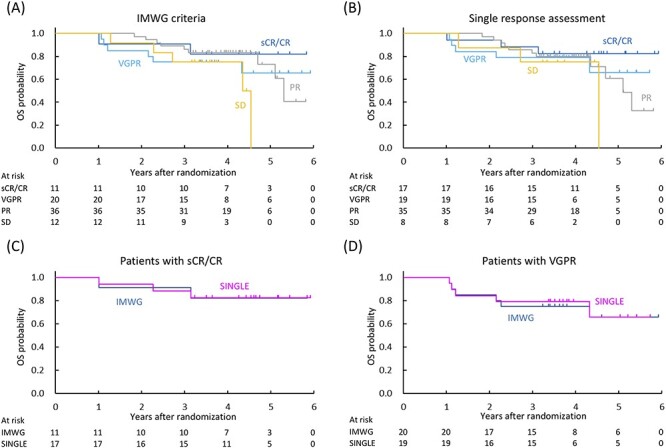
OS curves by response status evaluated using the IMWG criteria (A) and the single response assessment (B). Patients with sCR/CR evaluated using the IMWG criteria (*n* = 11) and the single response assessment (*n* = 17) (C). Patients with VGPR evaluated using the IMWG criteria (*n* = 20) and the single response assessment (*n* = 19) (D). OS, overall survival.

The 3-year OS according to the IMWG criteria was 90.9% (95% CI, 50.8–98.7) for sCR/CR, 75.0% (95% CI, 50.0–88.7) for VGPR, 86.1% (95% CI, 69.8–94.0) for PR and 75.0% (95% CI, 40.8–91.2) for SD (Fig. 3A). The 3-year OS according to the single response assessment was 88.2% (95% CI, 60.6–96.9) for sCR/CR, 78.9% (95% CI, 53.2–91.5) for VGPR, 82.9% (95% CI, 65.8–91.9) for PR and 75.0% (95% CI, 31.5–93.1) for SD (Fig. 3B). There was no significant difference in OS between the IMWG criteria and the single response assessment in the patients with sCR/CR or VGPR (Fig. 3C and D).

**Figure 4.**
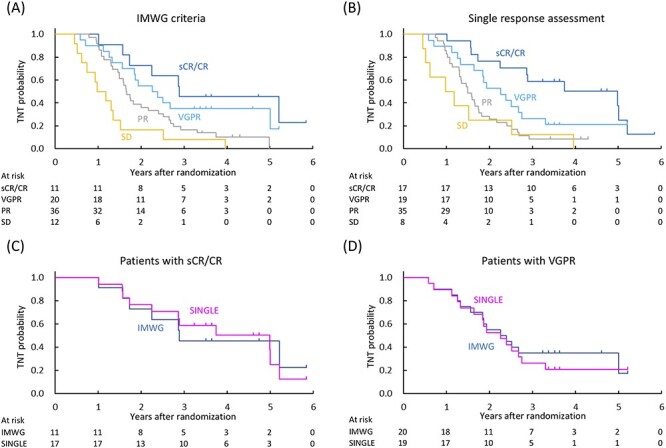
TNT by response status evaluated using the IMWG criteria (A) and the single response assessment (B). Patients with sCR/CR evaluated using the IMWG criteria (*n* = 11) and the single response assessment (*n* = 17) (C). Patients with VGPR evaluated using the IMWG criteria (*n* = 20) and the single response assessment (*n* = 19) (D). TNT, time to next treatment.

The 3-year TNT according to the IMWG criteria was 45.5% (95% CI, 16.7–70.7) for sCR/CR, 35.0% (95% CI, 15.7–55.2) for VGPR, 16.7% (95% CI, 6.8–30.4) for PR and 8.3% (95% CI, 0.5–31.1) for SD (Fig. 4A). The 3-year TNT according to the single response assessment was 58.8% (95% CI, 32.5–77.8) for sCR/CR, 26.3% (95% CI, 9.6–46.8) for VGPR, 8.6% (95% CI, 2.2–20.6) for PR and 12.5% (95% CI, 0.7–42.3) for SD (Fig. 4B). The median TNT in sCR/CR patients was 2.9 years (95% CI, 1.6—not estimable) by the IMWG criteria and 5.0 years (95% CI, 1.7–5.2) by the single response assessment (Fig. 4C). The median TNT in VGPR patients was 2.3 years (95% CI, 1.3–5.0) by the IMWG criteria and 2.3 years (95% CI, 1.3–2.8) by the single response assessment (Fig. 4D).

We also performed the same survival analysis for Arm A and Arm B. There was no difference in PFS (Supplemental Fig. S1), OS (Supplemental Fig. S2) or TNT (Supplemental Fig. S3) between IMWG criteria and single response assessment.

## Discussion

This explanatory analysis was performed to evaluate whether a single response assessment can substitute the current IMWG criteria using data from a randomized phase II study that developed a more promising MPB regimen for TI-NDMM (JCOG1105). Herein, we demonstrated that the single response assessment may be used as a substitute for the current IMWG criteria with two consecutive assessments for TI-NDMM.

The IMWG published uniform response criteria for clinical trials (2). They eliminated the mandatory minimal 6-week interval to confirm the achievement of response because the 6-week response duration does not carry major clinical significance and is not a surrogate for the durability of response. However, they required two consecutive assessments made at any time to eliminate laboratory or other errors. In 2016, the IMWG published new response criteria that retained two consecutive assessments of M protein levels (3). There have been no reports on the single response assessment of paraprotein levels and no discussion on whether two consecutive assessments were essential or not. The use of response criteria, which required two consecutive assessments, may underestimate the best response in the absence of a second assessment. Indeed, 13 patients (16.4%) of 79 in this analysis were not evaluated in a second response assessment. The best response for 5 out of the 13 patients was cycle 9 (the last cycle), and the IMWG criteria may underestimate the late responder because of the lack of a second evaluation. The SWOG S0777 randomized, open-label phase III trial on bortezomib, lenalidomide and dexamethasone (BLd) was reported (9), and the overall response rate to BLd regimen in that study was lower than that in the original BLd study (10) (81.5 vs. 100%). They discussed that one reason for these differences was that 20 patients (9%) in the BLd group who were not evaluated for the second response assessment had unconfirmed PR and were listed in the confirmed SD category.

Although careful judgment is required because M protein is the surrogate marker for abnormal plasma cells in MM patients, there is little evidence that two consecutive assessments are mandatory. Indeed, the level of agreement between the IMWG criteria and the single response assessment was sufficiently high (kappa coefficient of ≥0.7) in our analysis. Furthermore, results of PFS, OS and TNT showed no significant difference between the IMWG criteria and the single response assessment. Although survival analysis was performed to ensure whether the single response assessment was not inferior to the IMWG criteria due to the possibility of response overestimation, the single response assessment seemed to produce longer median PFS (3.8 vs. 2.9 years) and TNT (5.0 vs. 2.9 years) than the IMWG criteria in sCR/CR patients. Similar survival in patients achieving sCR/CR in the single response assessment compared with that in patients achieving CR in the more rigorous IMWG criteria is an important finding that supports the notion that single response assessment might be sufficient as long as there are no mistakes in the specimens.

Recent attempts had focused on the identification of residual tumor cells in the bone marrow using multi-color flow cytometry or next-generation sequencing (11). IMWG has defined new response categories of minimal residual disease (MRD) negativity, and there was no need for two consecutive assessments for MRD (3). MRD tests should be initiated only at the time of a suspected CR. Since sCR/CR of the single response assessment increased from 13.9 to 21.5% compared with those in the IMWG criteria in this study, the single response assessment may increase the chance of measuring MRD. The importance of MRD measurement is likely to increase in the future, and treatment stratification by MRD is being considered. When considering treatment strategies, such as shortening treatment in the MRD-negative patients, the single response assessment allows for more MRD testing to identify these patients.

There are several limitations in this supplementary analysis. First, JCOG1105 was not designed to analyze the new response criteria. Second, the number of patients analyzed was small. Finally, we could not conclude whether the single response assessment was also applicable to patients with transplant-eligible MM because all patients who were analyzed in this study were transplant-ineligible. However, to our knowledge, this is the first study addressing the utility of the single response assessment for MM. Recently, a suggestion for simplifying the IMWG criteria regarding the utility of repeating bone marrow biopsy for confirmation of CR was reported (12), and our study was also one of such attempts.

In conclusion, we found that the single response assessment could be a substitute for the current IMWG criteria with two consecutive assessments in patients with TI-NDMM. As this exploratory analysis included a limited number of patients, further investigation designed prospectively is necessary to confirm our results. We are also planning to validate the single response assessment for MM in the next phase III trial of JCOG–LSG (JCOG1911).

## Supplementary Material

JCOG1105S1_Supplementary_Figure_hyab066

JCOG1105S1_Table_S1_20210414_hyab066
