# Supplementary material for: Single response assessment of transplant-ineligible multiple myeloma: a supplementary analysis of JCOG1105 (JCOG1105S1)
Source: Jpn J Clin Oncol. 2021 May 6;51(7):1059–66. doi: 10.1093/jjco/hyab066 (PMC8246272; doi:10.1093/jjco/hyab066)
Supplement: JCOG1105S1_Supplementary_Figure_hyab066 [file jcog1105s1_supplementary_figure_hyab066.ppt]

## Slide 1
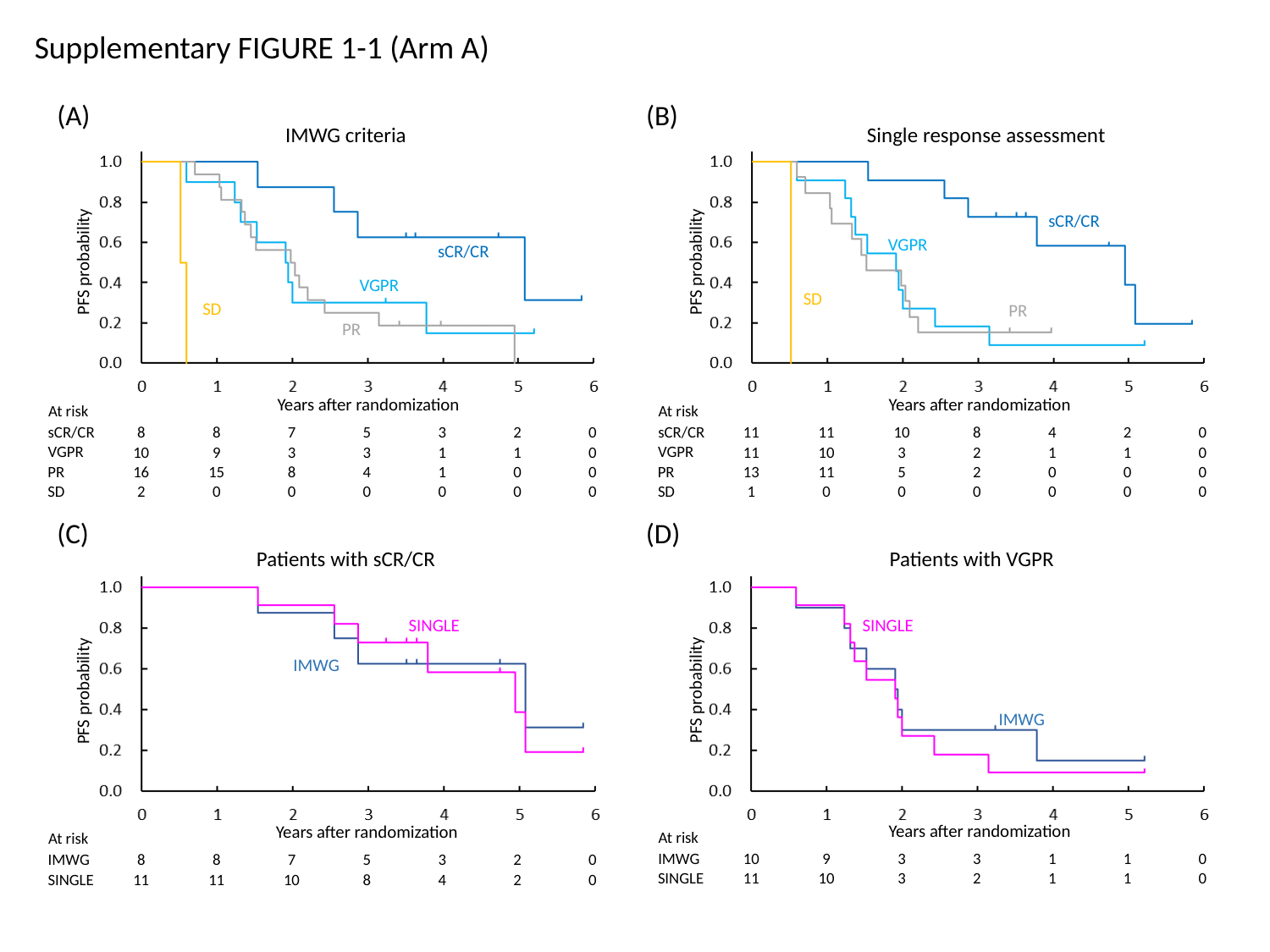

Supplementary FIGURE 1-1 (Arm A)
(A)
(B)
IMWG criteria
Single response assessment
sCR/CR
VGPR
sCR/CR
PFS probability
PFS probability
VGPR
SD
SD
PR
PR
Years after randomization
Years after randomization
At risk
8
8
7
5
3
2
0
sCR/CR
10
9
3
3
1
1
0
VGPR
16
15
8
4
1
0
0
PR
2
0
0
0
0
0
0
SD
At risk
11
11
10
8
4
2
0
sCR/CR
11
10
3
2
1
1
0
VGPR
13
11
5
2
0
0
0
PR
1
0
0
0
0
0
0
SD
(C)
(D)
Patients with sCR/CR
Patients with VGPR
SINGLE
SINGLE
IMWG
PFS probability
PFS probability
IMWG
Years after randomization
Years after randomization
At risk
10
9
3
3
1
1
0
IMWG
11
10
3
2
1
1
0
SINGLE
At risk
8
8
7
5
3
2
0
IMWG
11
11
10
8
4
2
0
SINGLE

## Slide 2
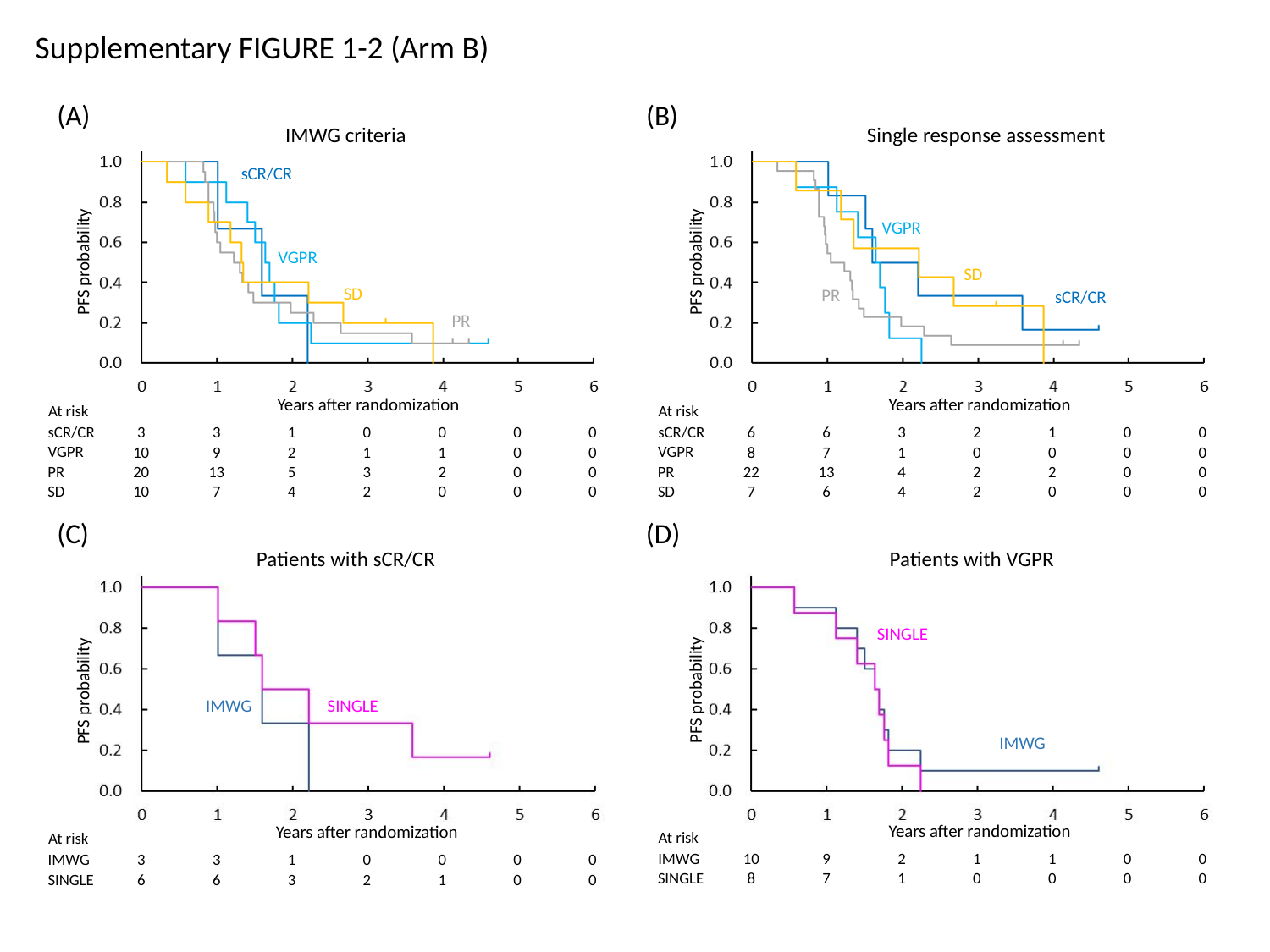

Supplementary FIGURE 1-2 (Arm B)
(A)
(B)
IMWG criteria
Single response assessment
sCR/CR
VGPR
VGPR
PFS probability
PFS probability
SD
SD
PR
sCR/CR
PR
Years after randomization
Years after randomization
At risk
3
3
1
0
0
0
0
sCR/CR
10
9
2
1
1
0
0
VGPR
20
13
5
3
2
0
0
PR
10
7
4
2
0
0
0
SD
At risk
6
6
3
2
1
0
0
sCR/CR
8
7
1
0
0
0
0
VGPR
22
13
4
2
2
0
0
PR
7
6
4
2
0
0
0
SD
(C)
(D)
Patients with sCR/CR
Patients with VGPR
SINGLE
PFS probability
PFS probability
SINGLE
IMWG
IMWG
Years after randomization
Years after randomization
At risk
10
9
2
1
1
0
0
IMWG
8
7
1
0
0
0
0
SINGLE
At risk
3
3
1
0
0
0
0
IMWG
6
6
3
2
1
0
0
SINGLE

## Slide 3
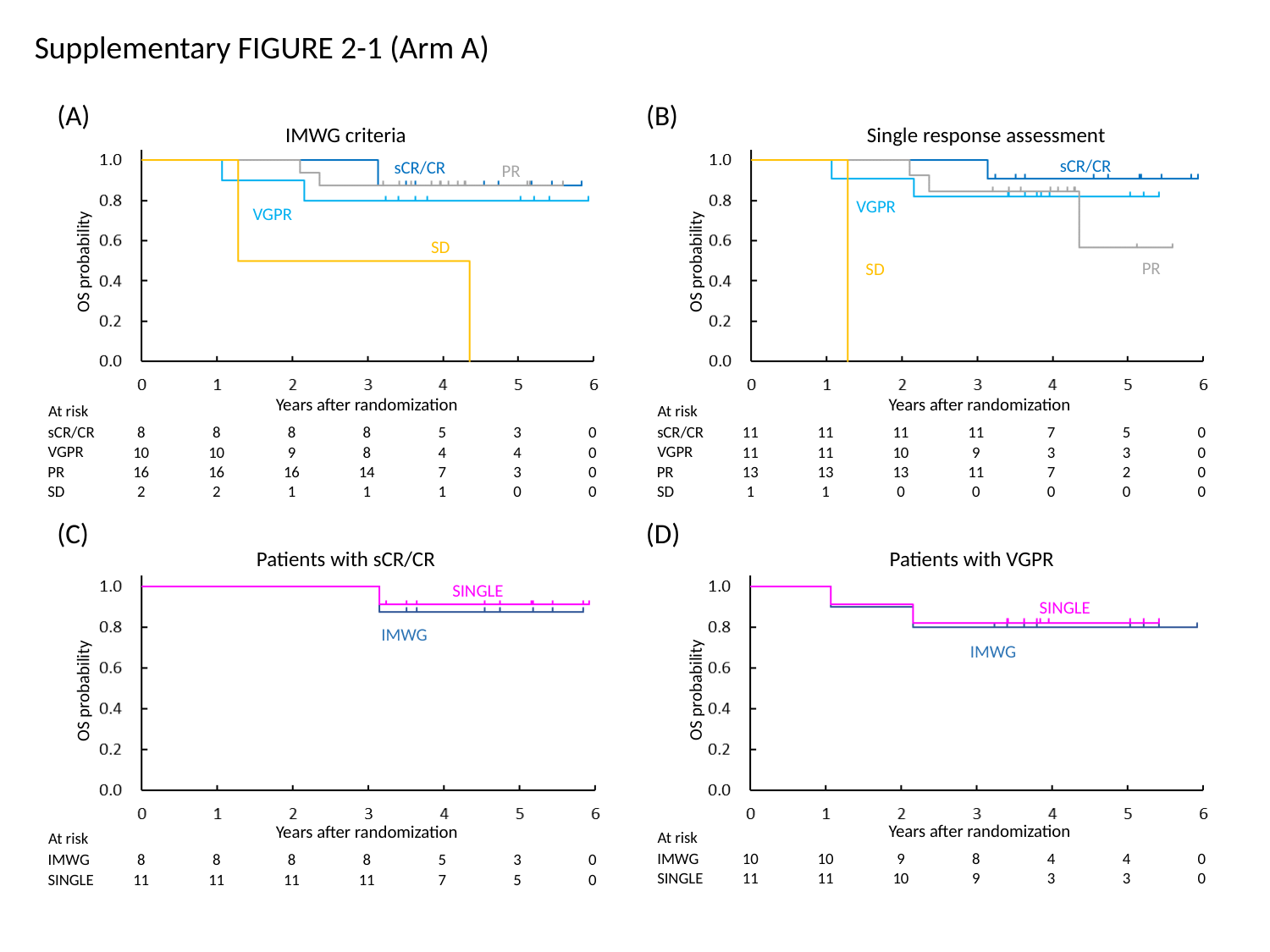

Supplementary FIGURE 2-1 (Arm A)
(A)
(B)
IMWG criteria
Single response assessment
sCR/CR
sCR/CR
PR
VGPR
VGPR
SD
OS probability
OS probability
PR
SD
Years after randomization
Years after randomization
At risk
8
8
8
8
5
3
0
sCR/CR
10
10
9
8
4
4
0
VGPR
16
16
16
14
7
3
0
PR
2
2
1
1
1
0
0
SD
At risk
11
11
11
11
7
5
0
sCR/CR
11
11
10
9
3
3
0
VGPR
13
13
13
11
7
2
0
PR
1
1
0
0
0
0
0
SD
(C)
(D)
Patients with sCR/CR
Patients with VGPR
SINGLE
SINGLE
IMWG
IMWG
OS probability
OS probability
Years after randomization
Years after randomization
At risk
10
10
9
8
4
4
0
IMWG
11
11
10
9
3
3
0
SINGLE
At risk
8
8
8
8
5
3
0
IMWG
11
11
11
11
7
5
0
SINGLE

## Slide 4
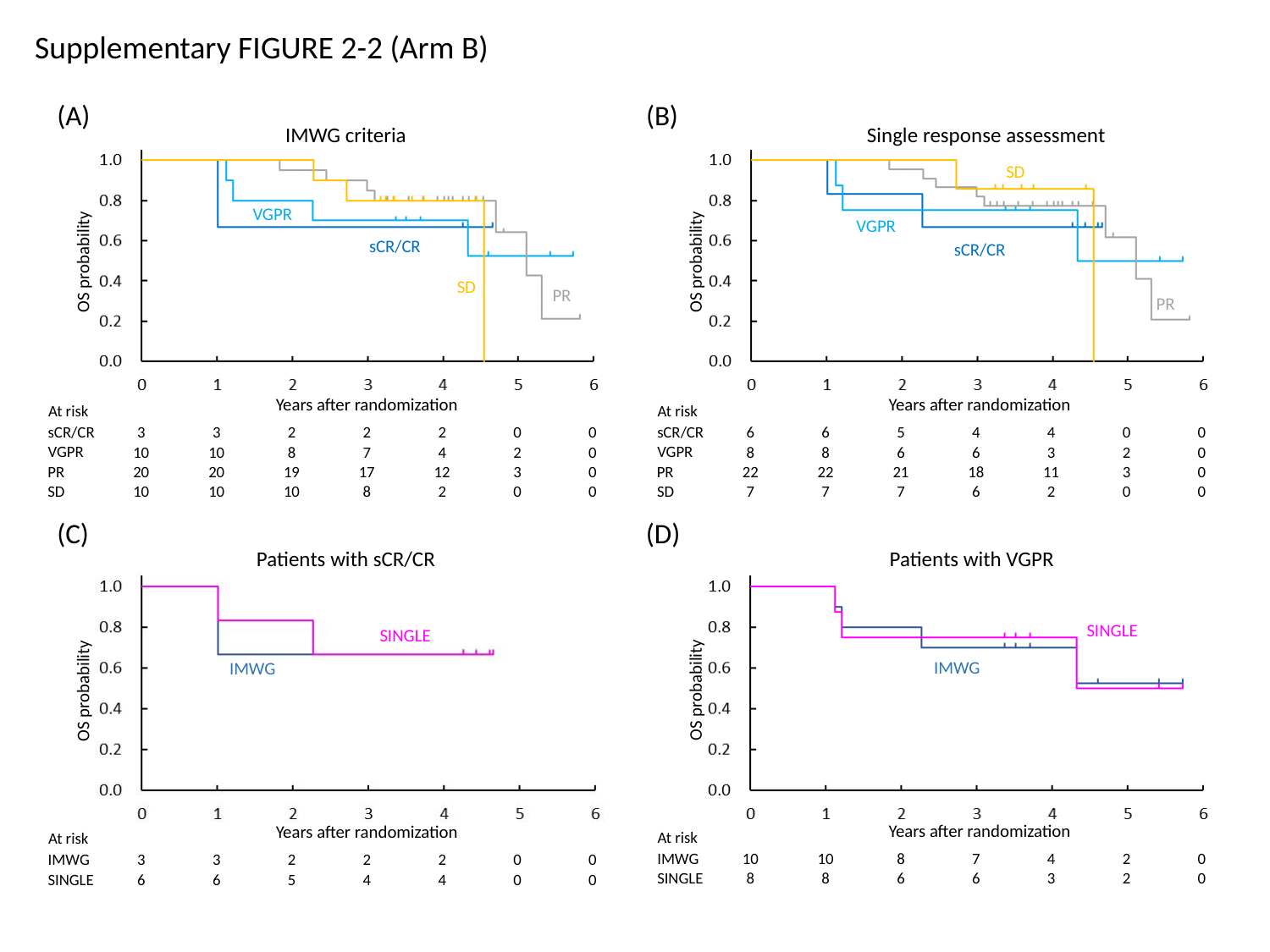

Supplementary FIGURE 2-2 (Arm B)
(A)
(B)
IMWG criteria
Single response assessment
SD
VGPR
VGPR
sCR/CR
sCR/CR
OS probability
OS probability
SD
PR
PR
Years after randomization
Years after randomization
At risk
3
3
2
2
2
0
0
sCR/CR
10
10
8
7
4
2
0
VGPR
20
20
19
17
12
3
0
PR
10
10
10
8
2
0
0
SD
At risk
6
6
5
4
4
0
0
sCR/CR
8
8
6
6
3
2
0
VGPR
22
22
21
18
11
3
0
PR
7
7
7
6
2
0
0
SD
(C)
(D)
Patients with sCR/CR
Patients with VGPR
SINGLE
SINGLE
IMWG
IMWG
OS probability
OS probability
Years after randomization
Years after randomization
At risk
10
10
8
7
4
2
0
IMWG
8
8
6
6
3
2
0
SINGLE
At risk
3
3
2
2
2
0
0
IMWG
6
6
5
4
4
0
0
SINGLE

## Slide 5
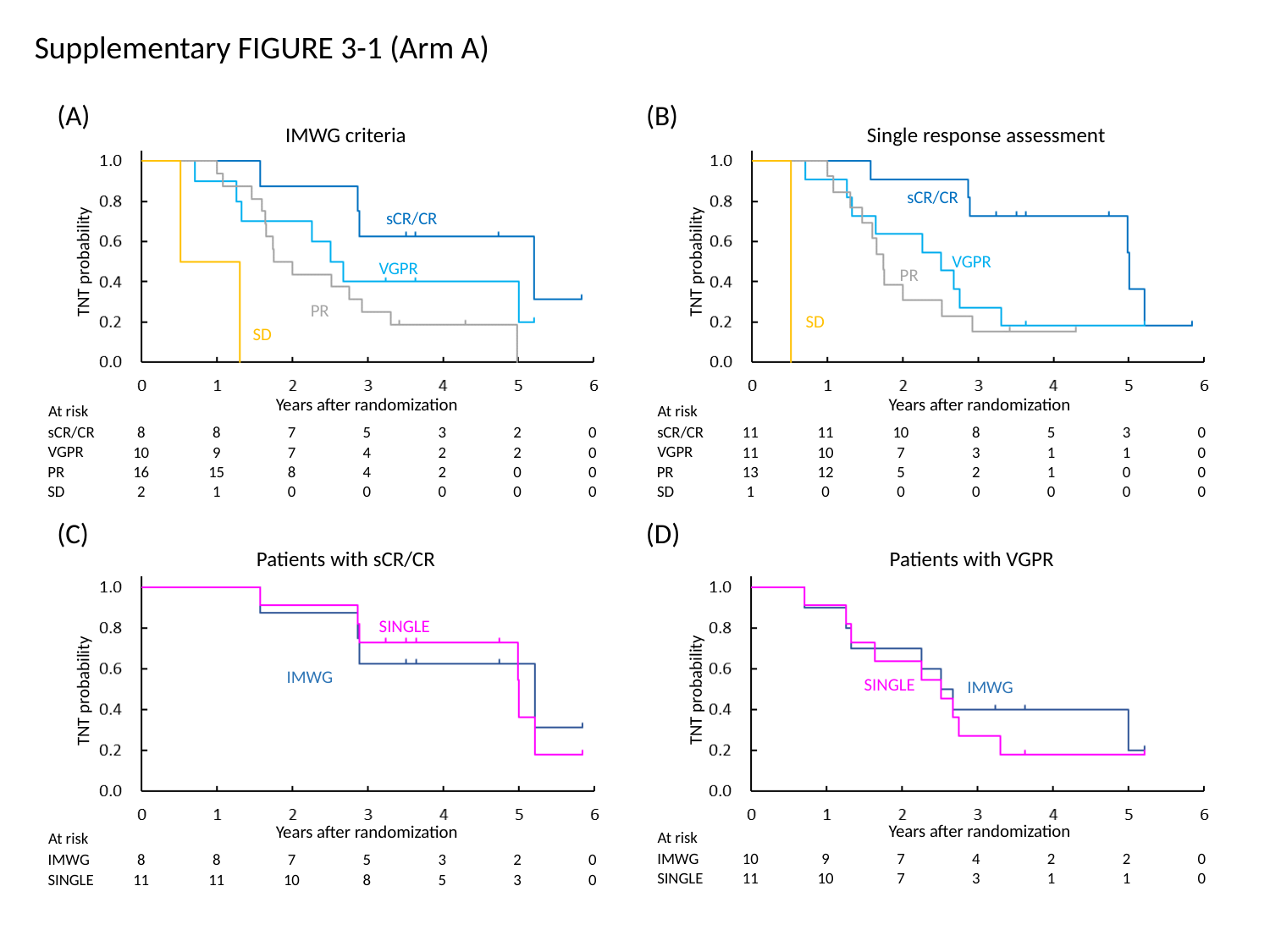

Supplementary FIGURE 3-1 (Arm A)
(A)
(B)
IMWG criteria
Single response assessment
sCR/CR
sCR/CR
VGPR
TNT probability
TNT probability
VGPR
PR
PR
SD
SD
Years after randomization
Years after randomization
At risk
8
8
7
5
3
2
0
sCR/CR
10
9
7
4
2
2
0
VGPR
16
15
8
4
2
0
0
PR
2
1
0
0
0
0
0
SD
At risk
11
11
10
8
5
3
0
sCR/CR
11
10
7
3
1
1
0
VGPR
13
12
5
2
1
0
0
PR
1
0
0
0
0
0
0
SD
(C)
(D)
Patients with sCR/CR
Patients with VGPR
SINGLE
IMWG
SINGLE
IMWG
TNT probability
TNT probability
Years after randomization
Years after randomization
At risk
10
9
7
4
2
2
0
IMWG
11
10
7
3
1
1
0
SINGLE
At risk
8
8
7
5
3
2
0
IMWG
11
11
10
8
5
3
0
SINGLE

## Slide 6
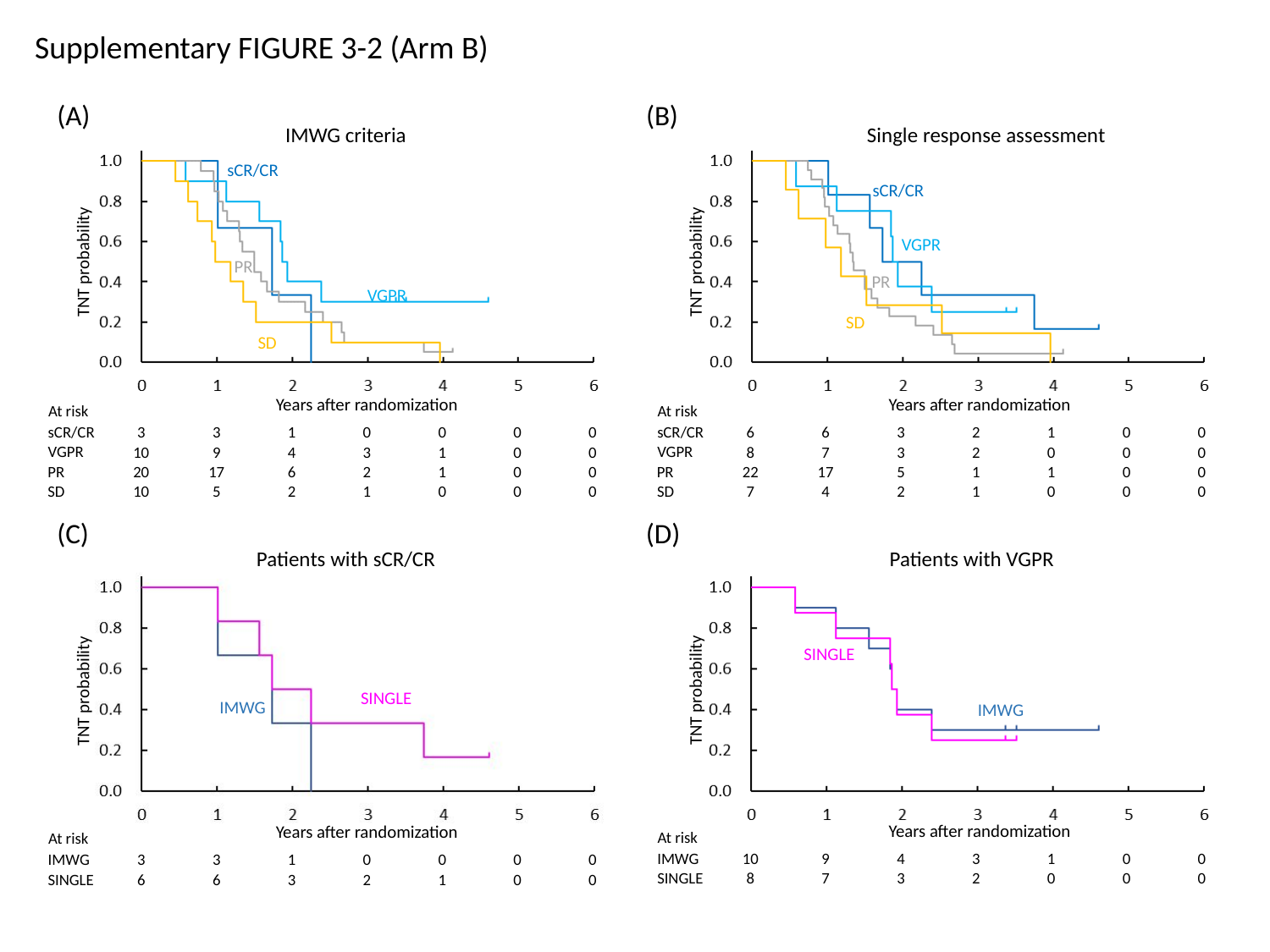

Supplementary FIGURE 3-2 (Arm B)
(A)
(B)
IMWG criteria
Single response assessment
sCR/CR
sCR/CR
VGPR
TNT probability
TNT probability
PR
PR
VGPR
SD
SD
Years after randomization
Years after randomization
At risk
3
3
1
0
0
0
0
sCR/CR
10
9
4
3
1
0
0
VGPR
20
17
6
2
1
0
0
PR
10
5
2
1
0
0
0
SD
At risk
6
6
3
2
1
0
0
sCR/CR
8
7
3
2
0
0
0
VGPR
22
17
5
1
1
0
0
PR
7
4
2
1
0
0
0
SD
(C)
(D)
Patients with sCR/CR
Patients with VGPR
SINGLE
TNT probability
TNT probability
SINGLE
IMWG
IMWG
Years after randomization
Years after randomization
At risk
10
9
4
3
1
0
0
IMWG
8
7
3
2
0
0
0
SINGLE
At risk
3
3
1
0
0
0
0
IMWG
6
6
3
2
1
0
0
SINGLE
